# New *Paenibacillus larvae* bacterial isolates from honey bee colonies infected with American foulbrood disease in Egypt

**DOI:** 10.1080/13102818.2014.906826

**Published:** 2014-07-08

**Authors:** Saad Hamdy Daif Masry, Sanaa Soliman Kabeil, Elsayed Elsayed Hafez

**Affiliations:** ^a^ Plant Protection and Biomolecular Diagnosis Department, Arid Lands Cultivation Research Institute, City of Scientific Research and Technology Applications, Alexandria, Egypt; ^b^ Protein Department, Genetic Engineering and Biotechnology Research Institute, City of Scientific Research and Technology Applications, Alexandria, Egypt

**Keywords:** American foulbrood disease, *Paenibacillus larvae*, honey bee (*Apis mellifera* L.), 16S rRNA

## Abstract

The American foulbrood disease is widely distributed all over the world and causes a serious problem for the honeybee industry. Different infected larvae were collected from different apiaries, ground in phosphate saline buffer (PSB) and bacterial isolation was carried out on nutrient agar medium. Different colonies were observed and were characterized biologically. Two bacterial isolates (SH11 and SH33) were subjected to molecular identification using 16S rRNA gene and the sequence analysis revealed that the two isolates are *Paenibacillus larvae* with identity not exceeding 83%. The DNA sequence alignment between the other *P. larvae* bacterial strains and the two identified bacterial isolates showed that all the examined bacterial strains have the same ancestor, i.e. they have the same origin. The SH33 isolate was closely related to the *P. larvae* isolated from Germany, whereas the isolate SH11 was close to the *P. larvae* isolated from India. The phylogenetic tree constructed for 20 different *Bacillus* sp. and the two isolates SH11 and SH33 demonstrated that the two isolates are *Bacillus* sp. and they are new isolates. The bacterial isolates will be subjected to more tests for more confirmations.

## Introduction

The American foulbrood (AFB) is the most serious disease of honeybee broods around the world and since it is capable of killing a colony, it causes considerable economic losses to beekeepers. The causative agent of AFB is the spore-forming bacterium *Paenibacillus larvae*,[1] whose only host is the honeybee (*Apis mellifera* L). Spores of *P. larvae* are the main vectors responsible for the spreading of the disease.[2] *P. larva* affects the larval and pupal stages of honeybee queens, workers and drones.[3,4] The number of spores required to infect a larva increases with larval age.[3,5,6] Sturtevant [7] reported that the infected larvae quickly die and about 2500 million spores form. Infected individuals turn brown and then black, and the resultant mass becomes a hard scale of material deposited on the side of the cell. In infected hives, *P. larvae* spores can be found not only in the brood but also in the honey, wax, pollen and hive walls.[8] *P. larvae* spores are transported among apiaries by drifting beehive parts, beekeepers’ clothes and contaminated pollen or honey, especially through common beekeeping practices and robbing diseased colonies.[9,10] It has been demonstrated that *P. larvae* spores are capable of germinating after 35 years in scales.[11,12]

Diagnosis of AFB is based on visual inspection of hives.[13] This procedure presents clear limitations because it depends on the judgement of an expert and relies on the observation of clinical symptoms that are not always easily recognized.[12,14] To confirm the visual AFB diagnosis, bacterial isolates need to be cultured and subsequently characterized morphologically, biochemically and physiologically.[15] Laboratory tests currently available are useful to confirm the presence of *P. larvae* in infected hives but do not allow epidemiological and surveillance studies.[16] In the case of infected pupae, the pupal tongue protrudes from the pupal head, extending to the top of the brood cell or may angle back towards the bottom of the cell. The protruding tongue is one of the most characteristic signs of the disease.[17]

Because of difficulties associated with AFB prevention and control,[16] AFB is subject to an official control program under the Biosecurity (National American Foulbrood Pest Management Strategy) Order 1998. Because of this, *P. larvae* will be classified as a hazard.[6] Foulbrood symptoms (possibly related to AFB) have been observed in recent field observations on Egyptian apiaries. There are very few reports about limited AFB infections in Egypt.[18] Abd Al-Fattah et al. [19] mentioned that AFB is a recent foe of honeybee colonies in Egypt and pointed out that the virulent nature of the bacterial pathogen, long and high rate of spore survival, with the wide range of infection routes dictate initiation of a control strategy with increasing the awareness amongst beekeepers about the early detection of AFB infections as well as the hygienic practices for restricting spread and control of this destructive disease.

Both Govan et al. [20] and Dobbelaere et al. [21] reported that polymerase chain reaction (PCR) could be used for rapid identification of *P. larvae*, by specific primers for different regions along the 16S rRNA gene. Govan et al. [20] designed primers that amplify a unique 973 bp amplicon, while Dobbelaere et al. [21] designed a combination of primers which succeeded to amplify four different single amplicons with molecular sizes of 970, 983, 1106 and 1119 bp. Martinez et al. [16] used real-time PCR as a tool for the detection of *P. larvae* vegetative cells and spores. Moreover, Chagas et al. [22] designed primers that amplify a 74 bp fragment and used these primers for the rapid detection of *P. larvae*. The aims of the present study were to isolate and identify Egyptian bacterial isolates that cause foulbrood disease, and plan a new strategy to control this disease in Egypt in case new bacterial isolates were discovered.

## Materials and methods

### Sample collection and diagnosis

**Figure 1.**
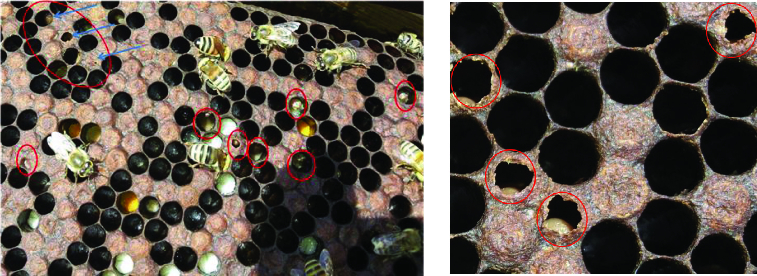
Irregular pattern of sealed brood with sunken and punctured caps typifying American foulbrood disease.

Two bee apiaries in Alexandria (Egypt), 30 colonies each, were inspected for honey bee diseases. AFB disease was diagnosed in the field according to the criteria of Shimanuki and Knox [23]. Brood combs showing severe symptoms of AFD were carefully collected from the infected apiaries during different seasons in 2012 and 2013. The brood was sampled by cutting out a piece comb of about 20 cm^2^ in size, containing as much of the dead or discoloured brood as possible.[24] Collected samples of larvae/pupae remains were obtained directly from the cells, significantly reducing the sample size and facilitating packaging, then kept at 4 °C [17,19] and immediately used for further laboratory diagnosis and isolation of the bacterial pathogen (Figure 1).

### Bacterial isolation

Pieces of comb of about 20 cm^2^ in size were ground in phosphate saline buffer (PSB) and different dilutions were performed. A 5 μL of each dilution was spread on Luria Broth (LB) antibiotic-free medium (Petri dishes). The plates were incubated at 30 °C overnight and the grown bacterial colonies were subjected to microscopic examination. One colony from each characterized bacterial isolate was subjected to molecular identification using the 16S rRNA gene.

### Bacterial DNA extraction

The bacterial genomic DNA was extracted using Wizard Genomic DNA purification kit (QIAGEN, Germany) according to the manufacturer's procedures.

### 16S rRNA amplification using specific PCR

The PCR reaction was performed according to Hafez and Elbestawy. [25] Primers 350 F and 350 R, corresponding to the polymorphic region of *E. coli*, 16S rRNA conserved gene sequence, respectively, forward: 5′ AGG ACG TGC TCC AAC CGC A 3′ and reverse 5′ AAC TGG AGG AAG GTG GGG AT 3′ were used to amplify approximately 350 bp of the 16S rRNA gene. The PCR reactions were performed in a total volume of 25 μL containing approximately 1 μL of genomic DNA (50 ng), 5 μL of 10X buffer, 3 μL of 2.5 mmol/L of dNTpase, 2.5 μL of MgCl_2_ (25 mmol/L), 2 μL of each primer (10 pmol/μL) and 0.2 U Taq DNA polymerase (Promega, USA). The PCR reaction conditions were: one cycle at 95 °C for 5 min; then 34 cycles of 95 °C for 1 min, 58 cycles for 1 min and 72 °C for 10 min; and a final extension cycle at 72 °C for 10 min. The PCR product was visualized in a 1% agarose gel and photographed using a gel documentation system (1-6A, Taiwan).

### DNA sequencing of the amplified 16S rRNA gene

The DNA sequence was performed using automated DNA sequencing and terminator dye (Macrogen Company, Korea).

### Sequence alignment and phylogenetic analysis

Pairwise and multiple DNA sequence alignment were carried out using the CLUSTALW multiple sequence alignment program version 1.82 (http://ww2.ebi.ac.uk/clustalw, [26]).

## Results and discussion

**Figure 2.**
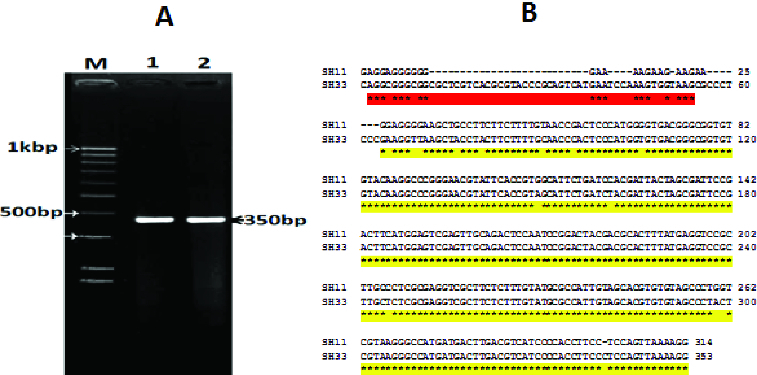
Visualization of PCR amplification products (A) and DNA nucleotide sequence (B) of the 16S rRNA gene of the two selected bacterial isolates (SH11 and SH33). Lane M: 1 KBP DNA marker; Lane 1: isolate SH11; Lane 2: isolate SH33.

The bacterial isolates were examined by microscope and stained with Gram stain. The colonies showed branched morphotype, 6 cm in diameter, dark red in colour and the cells were Gram-positive. To prove whether the isolates are *P. larvae*, PCR analysis was performed. Only two bacterial isolates were selected based on their location. The PCR amplification of the two morphologically characterized bacteria gave a fragment with a molecular size of 359 bp (Figure 2). The sequence analysis revealed that the two bacterial strains are *P. larvae*, and the sequences were deposited in gene bank (accession numbers SH11: KF724891 and SH33: KF724892). The amplified 359 bp fragment belongs to the variable region in the 16S rRNA gene, which is a good, easy method for bacterial identification.[27] Chagas et al. [22] succeeded to detect 29 different *P. larvae* strains by amplifying 70 bp from the 16S rRNA gene, using the real-time PCR. They reported that the real-time PCR of partial 16S rDNA gene of *P. larvae* represents an important alternative for rapid diagnosis of AFD. The results represented in this study are in agreement with that of Chagas et al.,[22] who suggested that the partial 16S rRNA PCR (real time) may represent an advancement for rapid confirmation of the presence of *P. larvae*.

**Figure 3.**
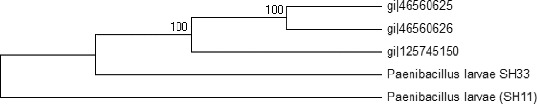
Phylogenetic tree of the two Egyptian bacterial isolates and the other three *P. larvae* bacterial strains based on the DNA nucleotide sequences of the 16S rRNA gene. The phylogeny was constructed by the Meg4 program (neighbour-joining tree).

The DNA-blast alignment showed that the obtained DNA sequences (320 bp) have similarity with only three bacterial strains of *P. larvae* subsp. *pulvifaciens* with 83% identity (gi|46560625, gi|46560626 and gi|125745150). This region is considered a variable region in the 16S rRNA gene of this bacterium (the region is located between base 1408 and 1168). It has been reported that the molecular diagnostic methods based on comparative analysis of sequences of the 16S rDNA gene are good tools for the detection and identification of *P. larvae.*[8,28] The comparative analysis based on the DNA nucleotide sequence of the two selected bacterial isolates revealed that isolate SH33 was closely related to the German strains (gi|46560625 and gi|46560626), whereas isolate SH11 tended to be closely related to the Indian one (gi|125745150). Similar results were obtained by Govan et al. [20] when they used the comparative study of the obtained DNA nucleotide sequence of the 16S rRNA gene to identify *P. larvae*. When the DNA nucleotide sequences of the two isolates (SH33; KF724892 and SH11; KF724891) were compared, the results showed the score of the alignment was about 75%. The evolutionary relationship between the two isolates and the previous three *P. larvae* strains was analysed and the results presented in Figure 3 illustrate the degree to which the two Egyptian isolates and the other three strains might have a common origin. In an earlier report, Alippi et al. [29] used the *ERIC* primers to amplify about 970 bp from the 16S rRNA gene and used the resultant DNA nucleotide sequence to design new primers that amplify about 550 bp. In our study, the SH33 isolate was grouped together with the other three strains, whereas isolate SH11 formed a separate group by itself, indicating that the two isolates are different in their genotype. These results agree with those of Genersch,[30] who postulated that there are four different genotypes of *P. larvae* based on PCR amplification using the *ERIC* specific primers. Moreover, the four genotypes of the *P. larvae* differed in their phenotype, including virulence and infection severity.[1,31,32]

**Figure 4.**
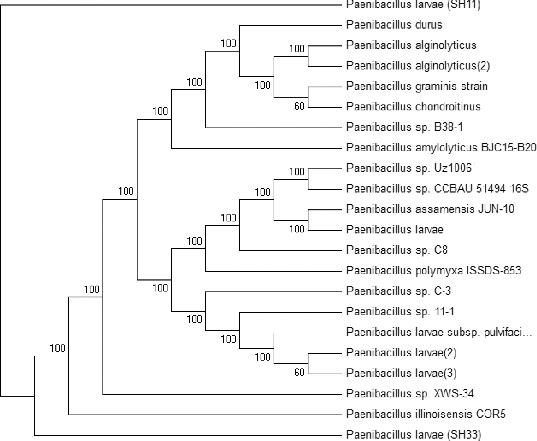
Phylogenetic tree of the two bacterial isolates *P. larvae* (SH33 and SH11) in comparison with 20 bacterial strains *Paenibacillus* sp. present in the gene bank. The phylogenetic tree was constructed based on the DNA nucleotide sequence of the 16S rRNA genes, using the Meg4 program (neighbour-joining tree).

A phylogenetic tree was constructed based on the two obtained DNA nucleotide sequences of the 16S rRNA genes and the sequences of 20 different *Paenibacillus* sp. The results presented in Figure 4 revealed that the 22 bacterial isolates were grouped into two main groups. Group 1 consists of 21 bacterial isolates and is divided into two main subgroups: subgroup 1 contains 19 different bacterial isolates and the second subgroup contains only one strain (SH33). Group 2 contains the SH11isolate only. The phylogenetic tree reflects the inferred evolutionary links of the two obtained bacterial isolates with the rest of the isolates. The results indicated that the two isolates and the other examined 20 isolates might have a distant common origin. We assume that the two bacterial isolates are new isolates based on all the previous analysis. Besides PCR, several other molecular methods such as single sequence repeat (SSR), inter single sequence repeat (ISSR), restriction fragment length polymorphism (RFLP) and sequencing combined with software [ClustalW, Mega6 etc.] permit the subtyping of the species *P. larvae.* Also, SDS-PAGE could be used for the characterization of the *P. larvae* profile [33] and this technique was used to classify subdivisions into clusters.[15]

## Conclusions

This study reports on new *P. larvae* bacterial isolates that infect honeybees in Egypt. This bacterial species is considered the main causal agent of AFB disease, which is a serious problem for the honeybee industry in Egypt. Strategies for biocontrol of this disease will be developed in the future.
